# A new insight into identification of in silico analysis of natural compounds targeting GPR120

**DOI:** 10.1007/s13721-018-0166-0

**Published:** 2018-05-14

**Authors:** Nagaraju Chinthakunta, Srinivasulu Cheemanapalli, Surekha Chinthakunta, C. M. Anuradha, Suresh Kumar Chitta

**Affiliations:** 1 Bioinformatics Infrastructure Facility, Department of Biochemistry, Sri Krishnadevaraya University, Anantapur, Andhra Pradesh 515003 India; 2 Department of Botany, Rayalaseema University, Kurnool, Andhra Pradesh 518007 India; 3 Department of Biotechnology, Sri Krishnadevaraya University, Anantapur, Andhra Pradesh 515003 India

**Keywords:** Colorectal cancer, G-protein coupled receptor 120, Homology modeling natural compounds, Rule of five, Docking studies, Potential therapeutics

## Abstract

G-protein coupled receptor (GPR120) is an omega-3 fatty acid receptor that inhibits macrophage-induced tissue inflammation. Recent studies revealed GPR120 promotes colorectal carcinoma through modulation of VEGF, IL-8, PGE2, and NF-kB expression. However, three-dimensional structure of GPR120 is not yet available in Protein Data Bank (PDB). In the present study, we focused on a 3-D structural model of GPR120 has been constructed using homology modeling techniques. The structural quality of the predicted GPR120 model was verified using Procheck, Whatif, ProSA, and Verify 3D. After this chemical database of natural compounds have been constructed and screened for its druggability using molinspiration server. Molecular docking studies of natural compounds on GPR120 model revealed that silibinin (− 6.87 kcal/mol), withanolide (− 6.19 kcal/mol), limonene (− 6.17 kcal/mol), and cervical (− 6.15 kcal/mol) have shown good docking interactions with active site residues of the target. Active site residues of Arg280, Asp275, and Gly122 showed hydrogen-bonding interactions with predicted compounds. Based on these in silico findings, we proposed that virtual screening of natural compounds against of GPR120 is a novel approach to find potential anti-colorectal cancer therapeutics.

## Introduction

Colon or colorectal cancer is a type of cancer that starts in the large intestine (colon) or the rectum (end of the colon). The origin of colon cancer is gastrointestinal tract of the epithelial cell lining present in the colon or rectum. Commonly mutations occur in the intestinal crypt cells (Ionov et al. 1993; Abdul Khalek et al. 2010). Colorectal cancer (CRC) is the third among most common malignancies worldwide (Shike et al. 1990) and the second leading cause of cancer deaths in the United States. It is estimated that 132,700 new cases were diagnosed in the United States in 2015 and 49,700 deaths occurred due to this disease (American Cancer Society 2015). Better medication is still a significant cause of cancer-associated deaths. CRC is based on a complex of diseases arising from multistep process events in enterocytes, including genetic, epigenetic events, and abnormal signaling in basic cellular pathways. Thus, it is a hallmark of clinical value to identify potential molecules for tumor-preventive strategies (Roberta Bertorelle et al. 2014).

In G-protein-coupled receptors (GPCRs), ligands bind specifically to GPCRs to stimulate and induce a variety of cellular responses via several second messenger pathways; e.g., modulation of cyclic-AMP production, the phospholipase C pathway, ion channels, and MAPK (Ulloa-Aguirre et al. 1999; Gether 2000; Schulte and Fredholm 2003). They are important signaling molecules for many aspects of cellular functions, including vision, olfaction, behavior, and autonomic transmission nervous system (Morris and Malbon 1999). Besides, they also regulate many characteristic features of tumorigenesis, including proliferation, invasion, survival at the secondary site, and immune cell function, as well as several cancer-associated signaling pathways (Feigin 2013). These properties permitted the widespread development of GPCR-targeted drugs, which represent nearly 30% of all currently used therapeutics (Lappano and Maggiolini 2011; Dorsam and Gutkind 2007). In particular, G-protein-coupled receptor 120 (GPR120), the most enigmatic member of this large family, has generated attention because of its potential role in the regulation of metabolic and inflammatory diseases such as obesity and type 2 diabetes.

G-protein coupled receptor 120 is a functional omega-3 FA receptor/sensor and mediates powerful insulin sensitizing and anti-diabetic effects by repressing macrophage-induced tissue inflammation (Oh et al. 2010). It is highly expressed in adipose tissue and proinflammatory macrophages, while activation of GPR120 affected LPS- and TNF-α-induced inflammatory signaling responses (Oh et al. 2010; Ichimura et al. 2012). Activation of GPR120 signaling induced the expression and secretion of proangiogenic mediators of CRC cells which promoted the angiogenesis. The PI3K/Akt–NF-kB pathway is activated by GPR120 signaling and required for GPR120 signaling-induced angiogenic switching in CRC cells. Furthermore, GPR120 activation enhanced motility of CRC cells and induced epithelial–mesenchymal transition (EMT) of CRC cells (Wu et al. 2013).

There is an increasing demand for natural compounds that improve human health. The World Health Organization estimated that approximately 80% of the world’s inhabitants rely on the traditional medicine for their primary health care (Farnsworth et al. 1985). Plants have long been used in the treatment of cancer (Hartwell 1971), and many nutritive and non-nutritive phytochemicals with diversified pharmacological properties have shown promising responses for the prevention and/or intervention of various cancers (Surh 2003), These products, especially phytochemicals, have been extensively studies and have exhibited anti-carcinogenic activities by interfering with the initiation, development, and progression of cancer through the modulation of various mechanisms including cellular proliferation, differentiation, apoptosis, angiogenesis, and metastasis (Rajesh et al. 2015). We focused on construction of GPR120 model using in silico tools and refinement of structure by docking studies with natural compounds which are believed to help in understanding of structural features and the interactions of natural compounds with GPR120 which may be helpful in designing of novel inhibitors of colorectal cancer.

## Materials and methods

In the present study, all the calculations were performed in a workplace by AMD 64 bits dual processing hi end server machines. Molecular modeling tasks were performed with Modeller9v3; docking calculations were performed with AutoDock 4.0. Unless otherwise stated, default settings were used during all calculations.

### Sequence alignments

All the analysis was carried out by AMD 64 bits dual processing hi end server machines. The sequence of G-protein coupled receptor120 (gi: 82581671) was obtained from the National Centre for Biotechnology Information (NCBI. http://www.ncbi.nlm.nih.gov/). Local alignments were predicted using Blastp (Basic Local Alignment Search Tool) (Altshul et al. 1997) at the NCBI and the homologous entries were obtained from the protein data bank (Berman et al. 2000). The Blastp alignment was further refined using sequence alignments in the Clustal W/X 1.83 with default parameters (Thompson et al. 1994).

### 3D model construction

The Blastp alignment was used for homology modeling built in Modeller9v3 (http://www.salilab.org/modeller/9v3) which generated structures by applying spatial restraints. A bundle of 100 models from the random generation of the starting structure was calculated and subsequently the best model (with the low RMS value of superposition using Swiss-pdb viewer) (Guex and Peitsch 1997). To gain a better relaxation and much apart arrangement of the atoms, refinement was done on the built GPR120 model by energy minimization (EM). The stabilization was assessed by graphics visualization.

### Evaluation of the homology model

The stereochemical parameters of the energy minimized GPR120 model were assessed by Procheck (Laskowski et al. 1993), Whatif (Vriend 1990), Errat (Colovos and Yeates 1993), ProSA (Sippl 1993) and Verify 3D (Bowie et al. 1991; Luthy et al. 1992). Verify 3D was used to assess whether a primary sequence is compatible with the current 3D structural model. The compatibility between the amino acid side chains in the model is a validation criterion. Torsion angle restraints for the side chains of each amino acid in the predicted GPR120 model were determined using a Web server Predictor (Berjanskii et al. 2006). The predictor assigns an error in the predicted chi (*χ*) torsion angle and including grains by combining its confidence scores with predicted or identified secondary structures and local sequence identity. Secondary structural conformations for the developed GPR120 model were predicted by Pdbsum (Laskowski et al. 2005).

### Docking studies

#### Selection and screening of natural ligands

To fulfil the aim of constructing a novel ligand for GPR120, we selected a library of 100 molecules from the previous publications and browsing Internet. The selected library of ligands was tested for Lipinski’s rule of five using molinspiration server (Lipinski et al. 2001) for their ability to follow the rule of five. Auto Dock 4.0/ADT (Goodsell and Morris 1998) program was used to investigate ligand binding to structurally refined GPR120 model using a grid spacing of 0.375 Å and the grid points in *X*, *Y,* and *Z* axis were set to 60 × 60 × 60. The search was based on the Lamarckian genetic algorithm (Miyamoto and Kollman 1992; Oprea et al. 2001) and the results were analyzed using binding energy. For each ligand, a docking experiment consisting of 100 stimulations was performed and the analysis was based on binding-free energies and root-mean-square deviation (RMSD) values. Docking with natural Compounds was also performed onto GPR120 model with the same parameters and PMV 1.4.5 viewer was then used to observe the interactions of the docked compounds to the GPR120 model (Kitchen et al. 2004) and we submitted the developed 3D model of GPR120 to Protein Model Data Base (PMDB) (Castrignano et al. 2006), which maintains 3D models obtained by structure prediction methods.

## Results and discussion

### Sequence alignments

**Fig. 1:**
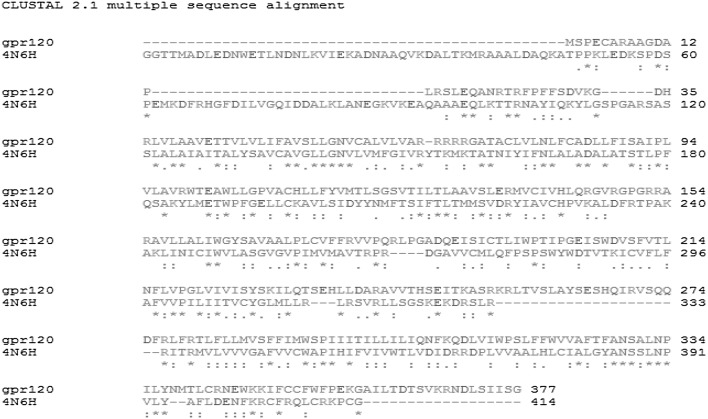
Multiple sequence alignment of GPR120 receptor and the template 4N6H. Highly conserved residues are represented by as stars

The coordinating 3D structure of Human Delta Opioid 7tm Receptor (PDB ID: 4N6H) (Fenalti et al. 2014). We found more than 70 crystallographic structures showing high identity score with respect to G-protein coupled receptor 120 using BLASTp results. We selected the Human Delta Opioid 7tm Receptor structure as template and the sequence identity between G-protein coupled receptor 120 and template 4N6H has 26% similarity having a resolution of 1.80 Å making it an excellent template. The most significant step in homology modeling process is to obtain the correct sequence alignment of the target sequence with the homologues. Finally, we performed an alignment between the selected template and the G-protein coupled receptor 120 using the ClustalX 1.8 with default parameters (Thompson et al. 1994). The sequence alignment performed homology modeling is shown in Fig. 1.

### Homology modeling

**Fig. 2:**
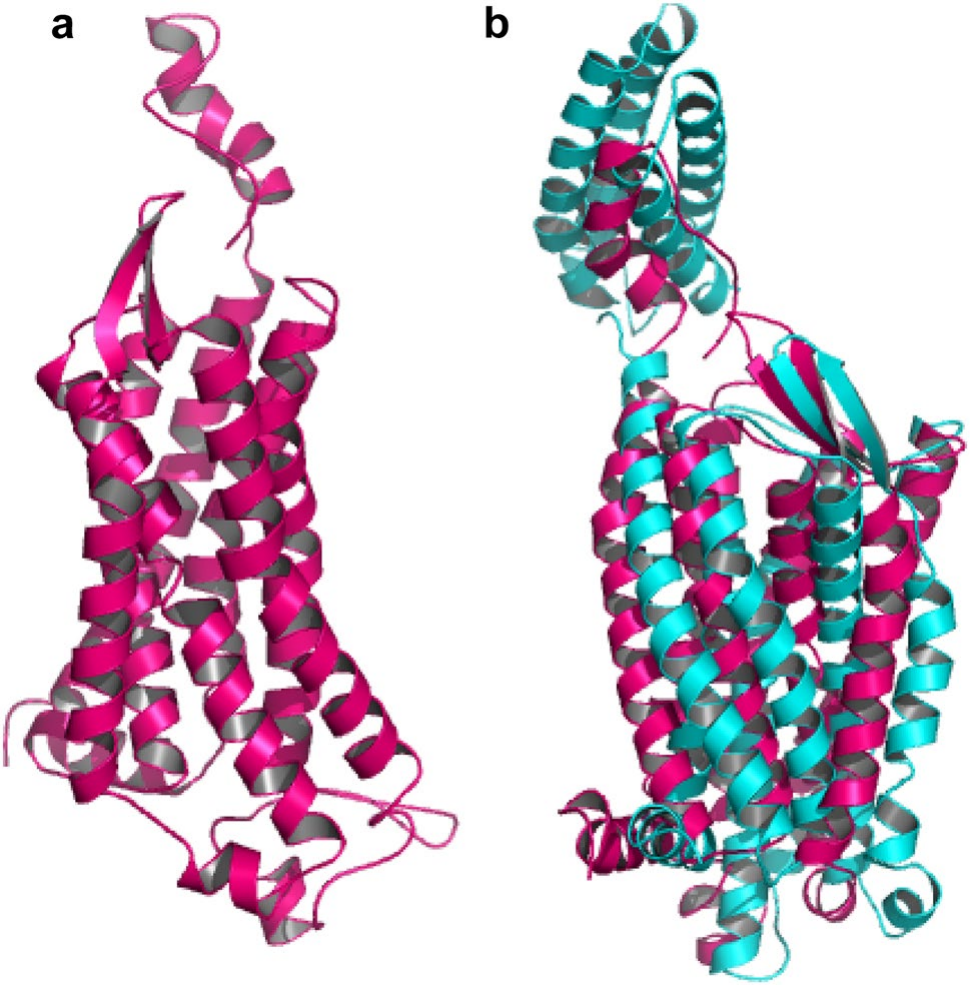
**a** Predicted 3-D structure of GPR120 using Modeller 9v3. **b** Superimposed structures of GPR120 (hot pink) and 4N6H (cyan)

The search using the BLASTp alignment algorithm within the PDB database showed various potential templates for molecular modeling purposes. More than 70 crystallographic structures showed high identity score with and maximum query coverage respect to G-protein coupled receptor. The coordinates of the crystal structures of Human Delta Opioid 7tm Receptor (PDB ID: 4N6H) (Castrignano et al. 2006) were used as a template to build the structure of G-protein coupled receptor 120. The 3D models of the G-protein coupled receptor 120 were built by Modeller 9v3. One hundred models were generated and the crystal structure of the template was saved for further refinement and validation (Fig. 2a, b). Furthermore, refinement was performed to obtain the best conformation of the developed model of G-protein coupled receptor 120.

### Structural validation of developed model

**Fig. 3:**
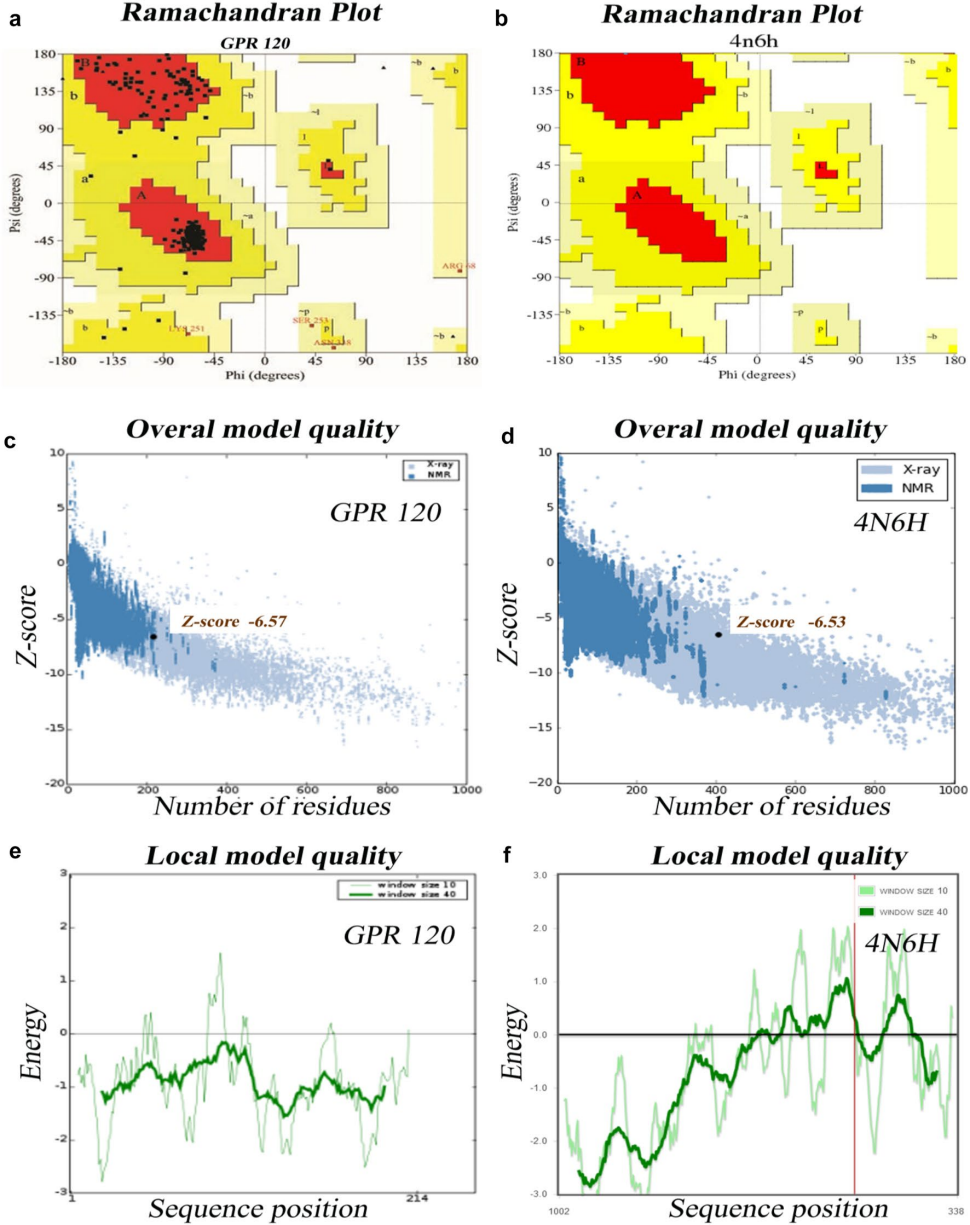
**a** ProSA-web Z-scores of all protein chains in PDB determined by X-ray crystallography (light blue) and NMR spectroscopy (dark blue) with respect to their length. The Z-score of GPR120 present in that range represented in large black dot. **b** Energy plot for the predicted GPR120

**Fig. 4:**
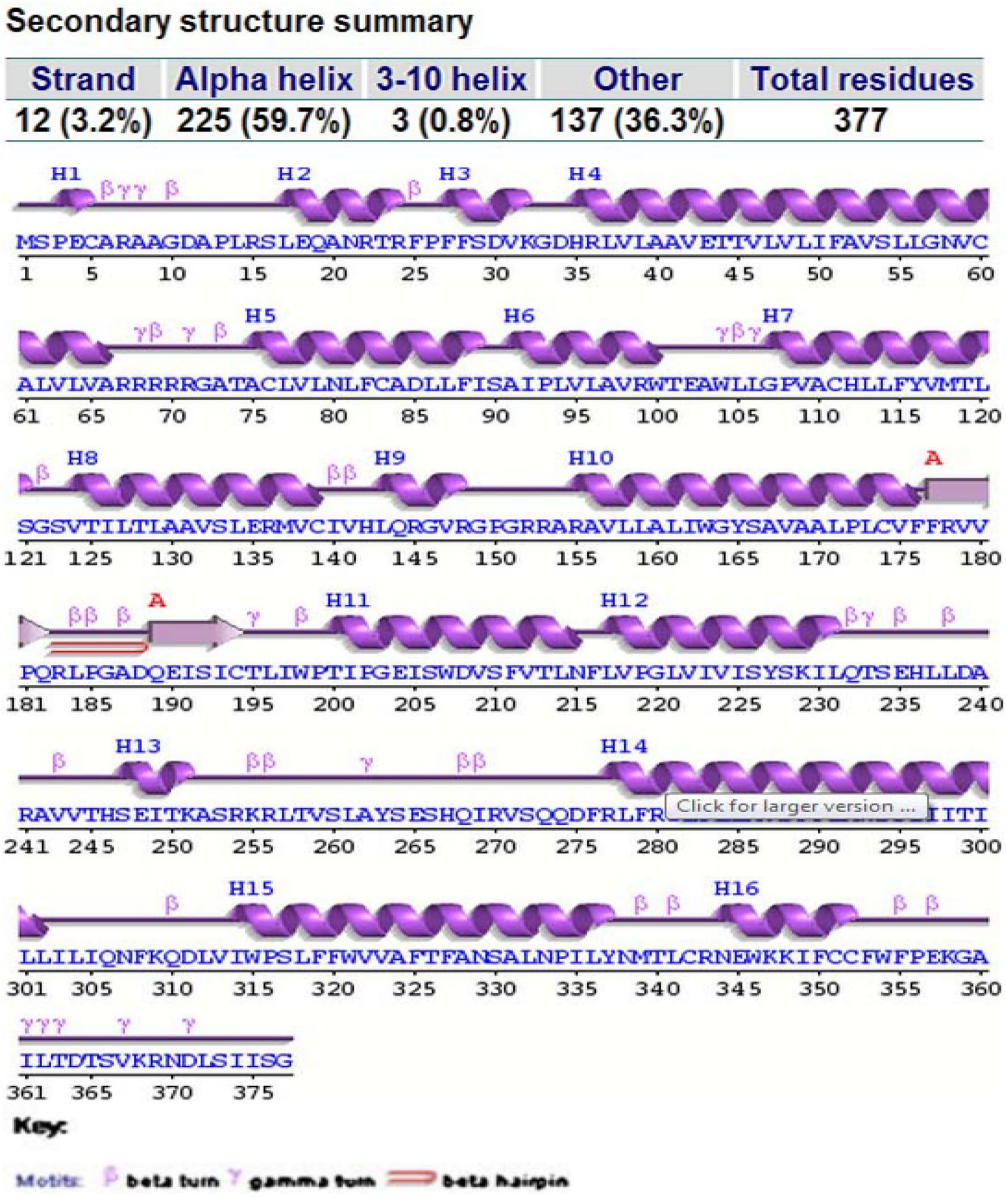
Secondary structure wiring diagram for the GPR120 showing the location of secondary structure elements

The constructed model was subjected to validation using Ramachandran plot with Procheck program by checking the detailed residue-by-residue stereochemical quality of a protein structure (Laskowski et al. 1993). The Ramachandran plot revealed that 100% of the residues in homology model were in favored and allowed regions. The main structural elements of the optimized GPR120 homology model are shown in Fig. 3. In comparison with the templates, the homology model had a similar Ramachandran plot with 0.0% residues in disallowed regions. The total quality G-factor was − 0.1, which indicates a good quality model (acceptable values of the G-factor in Procheck are between 0 and − 0.5, with the best models displaying values close to zero) showed in (Table 1). The Errat is a so-called “overall quality factor” for non-bonded atomic interactions and higher scores mean higher quality (Sippl 1993). The normally accepted range is > 50 for a high-quality model (Colovos and Yeates 1993). In the current case, the Errat score for the GPR120 model is 54.366, which well within the range of a high-quality model. Analysis of the energy minimized GPR120 model with Whatif web interface (Vriend 1990) revealed that RMS Z-Scores for bond angles and bond lengths are all close to 1 and also within the limits of template. Detailed structural investigation of the predicted GPR120 model with Pdbsum, a secondary structure prediction server, revealed that 225 (59.7%) residues are in α-helices and 3 (0.8%) residues are in 3–10 helix and 137 (36.3%) residues are in other conformations (Fig. 4, Laskowski et al. 2005). The tertiary structure of GPR120 showed close similarity to crystallized 4N6H, with a backbone RMS value of GPR120–4N6H, is between 0.58 and 0.72 Å, respectively. The low RMS values for backbone superposition reflect the high structural conservation of this complex through evaluation, making it a good system for homology modeling.

**Table 1:** Ramachandran plot statistics

Residues in most favored regions	328	95.1%
Residues in additional allowed regions	13	3.8%
Residues in generously allowed regions	4	1.2%
Residues in disallowed regions	0	0.0%
Number of non-glycine and non proline residues	345	100.00%
Number of end-residues (excl. Gly and Pro)	1	
Number of glycine residues (shown as triangle)	15	
Number of proline residues	16	
Total number of residues	377

### Screening and docking studies of natural inhibitors of GPR120

Docking is frequently used to predict the binding orientation of small molecule drug candidates to their protein targets to predict the affinity and activity of the small molecule. Hence, docking plays an important role in the rational design of drugs (Kitchen et al. 2004). Docking studies were performed to gain insight into the binding interaction between constructed model of GPR120 and selected 100 natural compounds.

#### Selection and screening of ligand molecules

One hundred natural compounds used as ligand molecules were taken from the National Centre for Biotechnology Information (NCBI) Pub-Chem database. These molecules were downloaded in Canonical SMILES format and converted to Protein Data Bank (PDB) coordinates file using Online SMILIS translate (http://cactus.nci.nih.gov/translate/). The selected ligand molecules were checked through the Molinspiration online server (http://www.molinspiration.com/cgi-bin/properties) for identifying their drug-likeness properties, and only 89 molecules that obey the Lipinski’s rule of five were used for further docking analysis (Table 2).

**Table 2:**
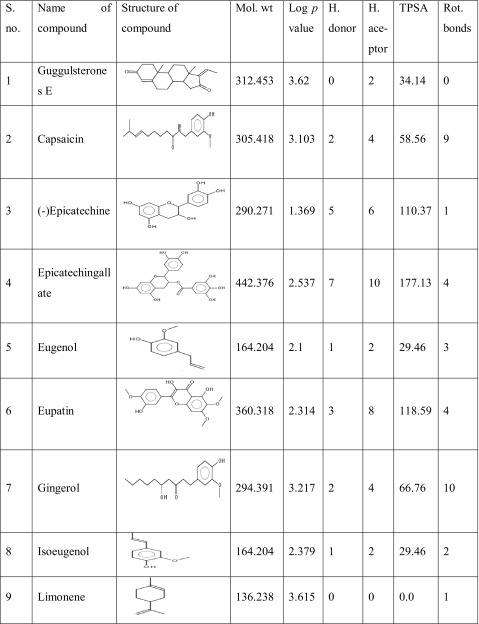

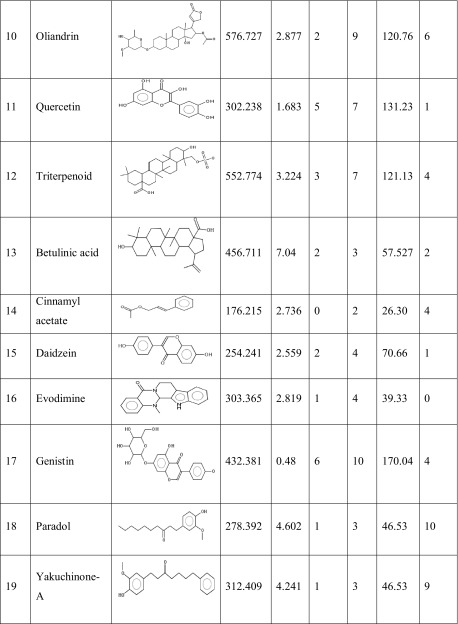

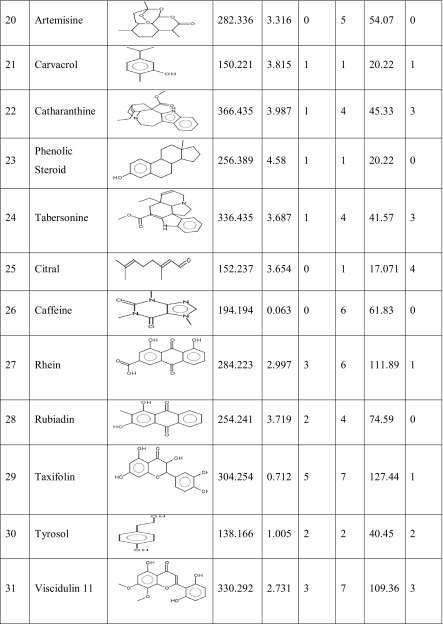

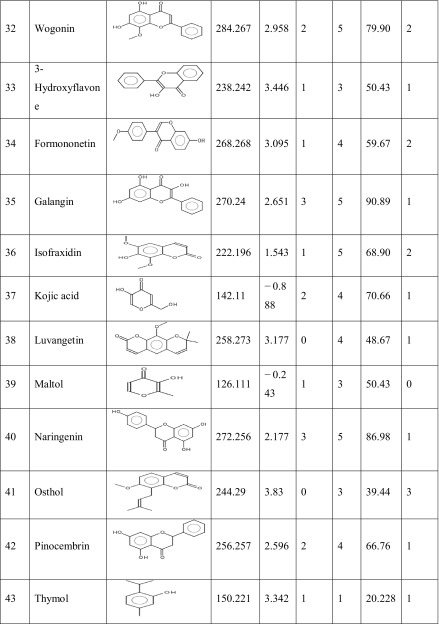

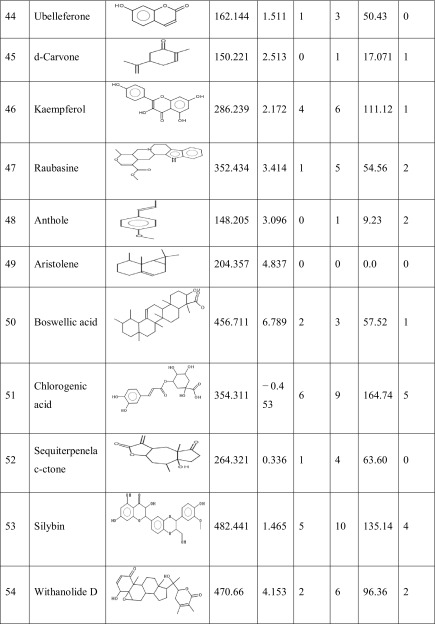

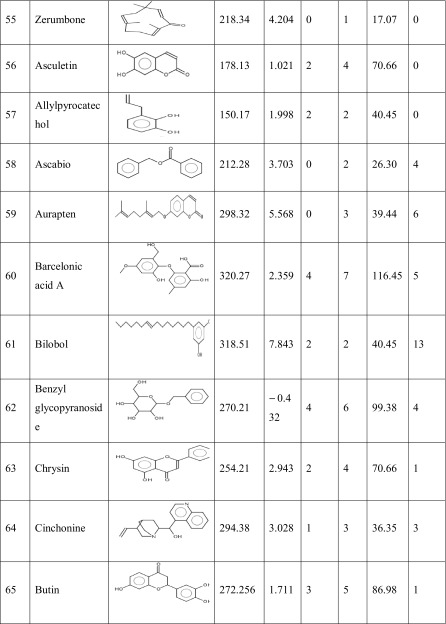

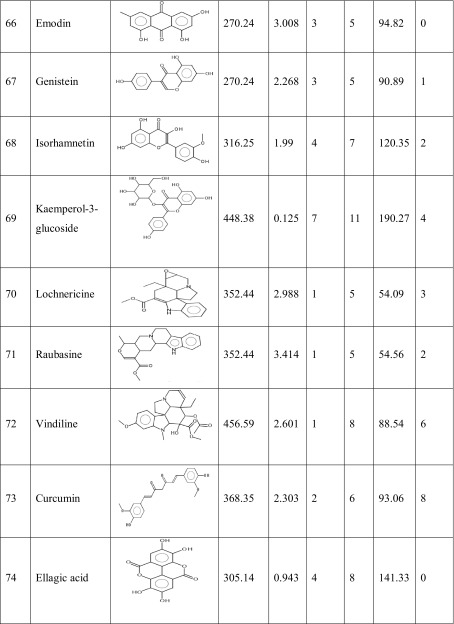

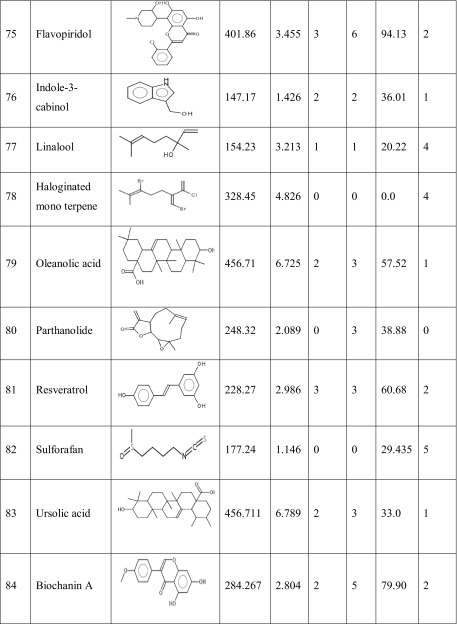

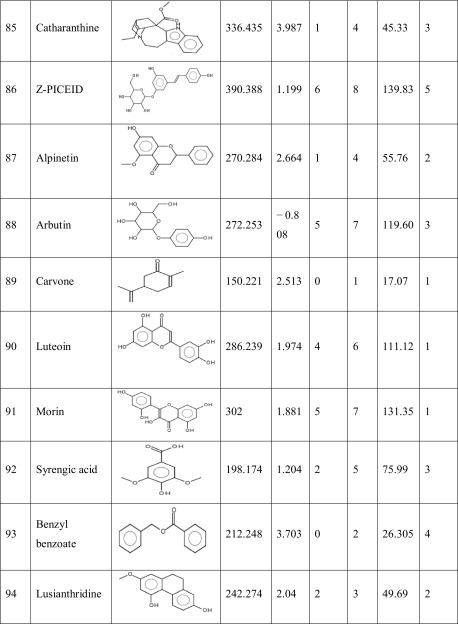

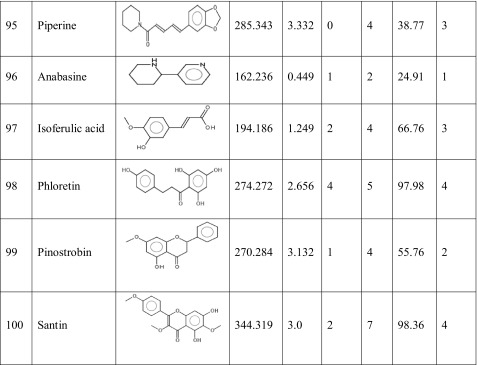
Drug-like properties of natural compounds used in docking studies onto G-protein coupled receptor 120

#### Docking studies of natural inhibitors with GPR120 model

**Fig. 5:**
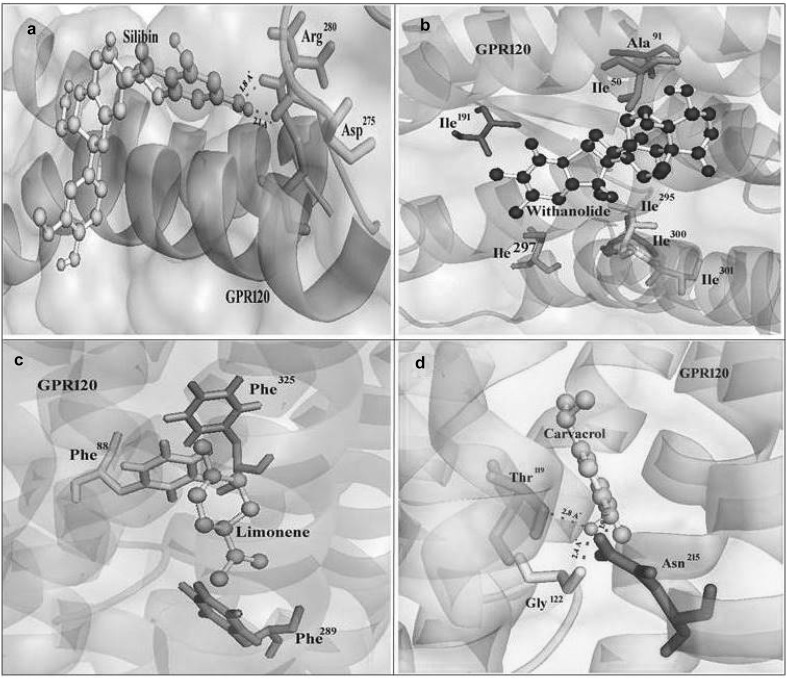
**a** Docking conformation of natural compound of Sylibin on G-protein coupled receptor 120 homology model. **b** Docking conformation of natural compound of Withanolide on G-protein coupled receptor 120 homology model. **c** Docking conformation natural compound of limonene on G-protein coupled receptor 120 homology model. **d** Docking conformation of natural compound of carvacrol on G-protein coupled receptor 120 homology models. Built model of G-protein coupled receptor 120 is represented in cartoon and 60% of electrostatic surface. Ligands are represented by ball and stick and the residues interacting with are represented by stics

Docking studies were performed to gain insight into the binding conformation of lead molecules with GPR120 model. A library of 100 lead molecules was constructed and screened for satisfying the minimal criteria of ADME for further analysis, using molinspiration. Among the 100 lead molecules, 89 molecules were selected based on the criteria of satisfying Lipinski’s rule of five with zero violations. All docking calculations were carried out using AutoDock 4.0/ADT and the dlg files generated were analyzed for their binding conformations. Analysis was based on free energy of binding, lowest docked energy, and calculated RMSD values (Table 3). The total clusters of docking conformations, with the 89 docked lead molecules, showed negative binding energies. Among all docking conformations, Silybin (Wing Ying Cheung et al. 2010), Withanolide D (Susmita Mondal et al. 2010), Limonene (Vigushin et al. 1998), and Carvacrol (Hailong et al. 2012), respectively, gave the best predicted binding-free energy of − 6.19, − 6.87, − 6.17, and − 6.15 kcal mol^−1^ to the GPR120 (Fig. 5, Table 4), and the corresponding references clearly uncloak which are all under clinical trails.

**Table 3:** Binding energies of docked natural compounds calculated by AutoDock

S. no.	Compound name	CID no.	Lowest binding energy (kcal/mol)	Inhibition constant (μM/mM)
1	Guggulsterones E	6439929	− 4.44	73.25
2	Capsacian	1548943	− 4.42	553.27
3	(-)Epicatechine	72276	− 4.57	447.16
4	Euenol	3314	− 3.8	308.86
5	Flavonoid	5317287	− 5.05	16.30
6	Gingerol	442793	− 3.86	20.13
7	Isoeugenol	853433	− 5.4	110.45
8	Limonene	22311	− 6.17	29.84
9	Quercetin	5280343	− 4.41	583.29
10	Cinnamyl acetate	5282110	− 6.05	36.67
11	Daidzein	5281708	− 5.25	141.64
12	Evodimine	151289	− 5.0	215.46
13	Paradol	94378	− 3.99	11.90
14	Yakuchinone-A	133145	− 4.35	651
15	Artemisine	68827	− 5.24	144.17
16	Carvacrol	10364	− 6.15	30.87
17	Catharanthine	197771	− 5.48	96.85
18	Phenolic steroid	439726	4.71	354.94
19	Tabersonine	20485	− 5.53	88.58
20	Citral	643779	− 4.95	235.43
21	Caffeine	2519	− 3.41	3190
22	Rhein	10168	− 4.7	359.53
23	Rubiadin	124062	− 4.53	477.98
24	Taxifolin	439533	− 4.68	373.32
25	Tyrosol	10393	− 4.63	400.67
26	Viscidulin 11	5322059	− 5.35	120.69
27	Wogonin	5281703	− 5.27	137.15
28	3-Hydroxyflavone	11349	− 5.07	192.3
29	Formononetin	5280378	− 4.39	608.88
30	Galangin	5281616	− 4.78	315.78
31	Isofraxidin	5318565	− 4.21	818.78
32	Kojic acid	3840	− 4.56	457.88
33	Luvangetin	343582	− 457	446.42
34	Maltol	8369	− 4.2	829.62
35	Naringenin	932	− 4.84	284.29
36	Osthol	10228	− 4.87	268.85
37	Pinocembrin	68071	− 5.25	141.93
38	Thymol	6989	− 3.5	320
39	Ubelleferone	5281426	− 5.98	41.05
40	d-Carvone	16724	− 5.63	75.18
41	Kaempferol	5280863	− 4.42	75.18
42	Raubasine	441975	− 5.13	173.58
43	Anthole	637563	− 4.1	991.37
44	Aristolene	530421	− 5.41	107.8
45	Sequiterpenelacctone	338659	− 5.58	81.2
46	Silybin	31553	− 6.87	9.12
47	Withanolide D	161671	− 6.19	78.71
48	Zerumbone	5470187	− 3.7	1860
49	Asculetin	5281416	− 4.51	496.78
50	Allylpyrocatechol	292101	5.09	185.5
51	Ascabiol	2345	− 4.51	496.78
52	Barcelonic acid A	10358625	− 3.73	18.60
53	Bilobol	5281852	− 2.97	1.86
54	Benzyl glycopyranoside	188977	− 4.24	782.76
55	Chrysin	5281607	− 4.81	297.75
56	Cinchonine	90454	− 4.89	260.15
57	Butin	92775	− 5.57	82.55
58	Emodin	3220	− 4.62	409.35
59	Genistein	5280961	− 5.64	73.25
60	Isorhamnetin	5281654	− 4.96	0.21
61	Kaemperol-3-glucoside	5282102	− 4.42	571.91
62	Lochnericine	11382599	− 5.19	156.7
63	Raubasine	441975	5.11	179.54
64	Vindiline	260535	− 5.92	45.74
65	Curcumin	969516	− 5.06	196.36
66	Flavopiridol	5287969	− 3.98	1210
67	Indole-3-cabinol	3712	− 3.53	2600
68	Linool	6549	3.53	2590
69	Haloginated mono terpene	11493622	− 4.45	544.66
70	Parthanolide	6473881	− 4.92	248.68
71	Resveratrol	445154	− 5.76	59.59
72	Sulforafan	5350	− 4.82	290.63
73	Biochanin A	5280373	− 4.96	232.7
74	Catharanthine	197771	− 5.58	0.22
75	Z-PICEID	10178463	− 5.72	63.73
76	Alpinetin	4053302	− 4.92	245.55
77	Arbutin	440936	− 5.04	202.08
78	Carvone	7439	− 5.27	137.55
79	Luteoin	5280445	− 5.76	60.28
80	Morin	5281670	− 4.3	705.91
81	Syrengic acid	10742	− 3.27	4030
82	Benzyl benzoate	2345	− 6.41	19.92
83	Lusianthridine	442702	− 3.72	1.88
84	Piperine	638024	− 4.59	432.06
85	Anabasine	2181	− 5.69	67.14
86	Isoferulic acid	736186	− 5.8	80.97
87	Phloretin	4788	− 3.72	1890
88	Pinostrobin	73201	− 5.29	132.4
89	Santin	5281695	− 4.91	251.48

**Table 4:** High score-binding energies of docked natural compounds calculated by AutoDock

Docked molecule	Compound Cid no.	Cluster rank	Cluster number	Binding energy (kcal/mol)	RMSD (Å)
Silybin	31553	1	3	− 6.87	0.49
Withanolide D	161671	1	3	− 6.19	0.82
Limonene	22311	2	41	− 6.17	0.13
Carvacrol	10364	1	19	− 6.15	0.65

To confirm the binding mode of natural substrate, docking was performed on the GPR120 model; natural substrate docking revealed that the amino acids Ile50 in orange, Phe88 in yellow, Ala91 in salmon, Gly122 in green, Ile191 in red, Asn215 in magenta, Asp275 in cyan, Arg280 in orange, Phe289 in salmon, Ile295 in yellow, Ile297 in spiltpea, Ile300 in salmon, Ile301 in yellow, and Phe325 in deepsalmon color (Fig. 5) played vital role to in binding the natural substrates and except Asn215, Asp275, and Arg280 all hydrophobic amino acids.

## Conclusion

In this study, we have developed a three-dimensional structure of GPR120 receptor through homology modeling using delta opioid 7tm receptor (PDB ID: 4N6H) as a template. The generated model was assessed by several validation tools like Procheck, Errat, whatif, ProSA 2007, and Verify 3D. All above-mentioned tools revealed that the model is reliable. This model was also submitted to PMDB server (PDB: PM0079568) for public assessment. From the available scientific literature, 100 natural compounds have been selected and 89 compounds followed the rule of five. These 89 natural compounds were docked with GPR120 receptor and the following four compounds have exhibited the highest binding energy levels in the order as CID: 31553 > CID: 161671 > CID: 22311 > CID: 10364, which infers the favorable rank score, docking score, and hydrogen-bonding energies. Furthermore, more hydrophobic interactions were observed in the binding pocket of the GPR120. These four drugs are under clinical trials and thereby help promising therapeutics for colorectal cancer.
